# Biochemical Targets and Molecular Mechanism of Ginsenoside Compound K in Treating Osteoporosis Based on Network Pharmacology

**DOI:** 10.3390/ijms232213921

**Published:** 2022-11-11

**Authors:** Sen Zhang, Shihong Shen, Pei Ma, Daidi Fan

**Affiliations:** 1 Shaanxi Key Laboratory of Degradable Biomedical Materials, School of Chemical Engineering, Northwest University, Xi’an 710069, China; 2 Shaanxi R&D Center of Biomaterials and Fermentation Engineering, School of Chemical Engineering, Northwest University, Xi’an 710069, China; 3 Biotech. & Biomed. Research Institute, Northwest University, Xi’an 710069, China

**Keywords:** ginsenoside CK, osteoporosis, network pharmacology, osteoblast differentiation pathway, c-Fms signaling

## Abstract

To investigate the potential of ginsenosides in treating osteoporosis, ginsenoside compound K (GCK) was selected to explore the potential targets and mechanism based on network pharmacology (NP). Based on text mining from public databases, 206 and 6590 targets were obtained for GCK and osteoporosis, respectively, in which 138 targets were identified as co-targets of GCK and osteoporosis using intersection analysis. Five central gene clusters and key genes (STAT3, PIK3R1, VEGFA, JAK2 and MAP2K1) were identified based on Molecular Complex Detection (MCODE) analysis through constructing a protein–protein interaction network using the STRING database. Gene Ontology (GO) analysis implied that phosphatidylinositol-related biological process, molecular modification and function may play an important role for GCK in the treatment of osteoporosis. Function and Kyoto Encyclopedia of Genes and Genomes (KEGG) analysis suggested that the c-Fms-mediated osteoclast differentiation pathway was one of the most important mechanisms for GCK in treating osteoporosis. Meanwhile, except for being identified as key targets based on cytoHubba analysis using Cytoscape software, MAPK and PI3K-related proteins were enriched in the downstream of the c-Fms-mediated osteoclast differentiation pathway. Molecular docking further confirmed that GCK could interact with the cavity on the surface of a c-Fms protein with the lowest binding energy (−8.27 Kcal/moL), and their complex was stabilized by hydrogen bonds (Thr578 (1.97 Å), Leu588 (2.02 Å, 2.18 Å), Ala590 (2.16 Å, 2.84 Å) and Cys 666 (1.93 Å)), van der Waals and alkyl hydrophobic interactions. Summarily, GCK could interfere with the occurrence and progress of osteoporosis through the c-Fms-mediated MAPK and PI3K signaling axis regulating osteoclast differentiation.

## 1. Introduction

Osteoporosis is a metabolic and systemic bone disease characterized by a loss of bone mass and the micro-architectural deterioration of bone tissue, susceptibly resulting in bone fragility and fracture [1,2]. Osteoporosis causes over 8.9 million fractures worldwide each year, most of which are located in the hip, spine, distal forearm, and proximal humerus [3]. With the progressive aging of the global population, the incidence of osteoporotic fractures with high mortality and morbidity is increasing dramatically [4]. It is reported that the morbidity of hip fractures will increase by 3.5 times between 1990 and 2050 all over the world [5]. Unfortunately, there are no drugs available for the effective treatment of osteoporosis at present. In the current clinical management of osteoporosis, relieving bone fractures is the major aim of drugs, of which the most common are bisphosphonates, such as alendronate, risedronate, zoledronic acid, ibandronate, and so on [6,7]. However, there are several limitations for bisphosphonate drugs, including acute renal failure, gastrointestinal intolerability, musculoskeletal pain, and in rare cases, an increased risk of fracture upon their long-term use, particularly of atypical femoral fractures and osteonecrosis of the jaw [7,8]. Therefore, discovering new effective drugs with less side effects for the treatment of osteoporosis has been an urgent requirement.

Natural products are important and reliable resources for the prevention and treatment of osteoporosis because they have fewer side effects and are more suitable for long-term application, compared with chemosynthetic medicines [9]. Ginseng, the root *of Panax ginseng* C.A. Meyer, has been used as a tonic remedy for more than 2000 years in Asia [10]. Ginsenosides are the main pharmacologically active compounds in ginseng, of which pharmacological effects include resistance to tumors, the inhibition of neurodegeneration in patients with Alzheimer’s disease, the promotion of brain development and memory improvement, the exhibition of anti-inflammatory and antioxidant effects, the prevention of diabetes, resistance to fatigue, the protection of the heart and anti-osteoporosis, etc. [11]. Ginsenoside Rb1, Rb2, Rg1, Rg3 and Rh2 can prevent osteoporosis based on in vitro or/and in vivo experiments [10,12,13,14]. However, the molecular mechanism is not totally understood for ginsenosides to prevent and treat osteoporosis. Ginsenoside compound K (GCK) (20-O-β-D-glucopyranosyl-20(S)-protopanaxadiol) does not naturally exist in ginseng, but it is the major metabolite of natural ginsenoside Rb1, Rb2 and Rc in the intestine under the effects of intestine bacteria [15]. GCK is considered a rare kind of ginsenoside and has received more and more attention because of its superior solubility, bioavailability, and bioactivity compared with its parent ginsenosides [15,16]. Therefore, GCK may also have more beneficial effects on the prevention and treatment of osteoporosis than its parent ginsenosides. However, investigations into the role of GCK in treating osteoporosis are relatively poor.

**Figure 1:**
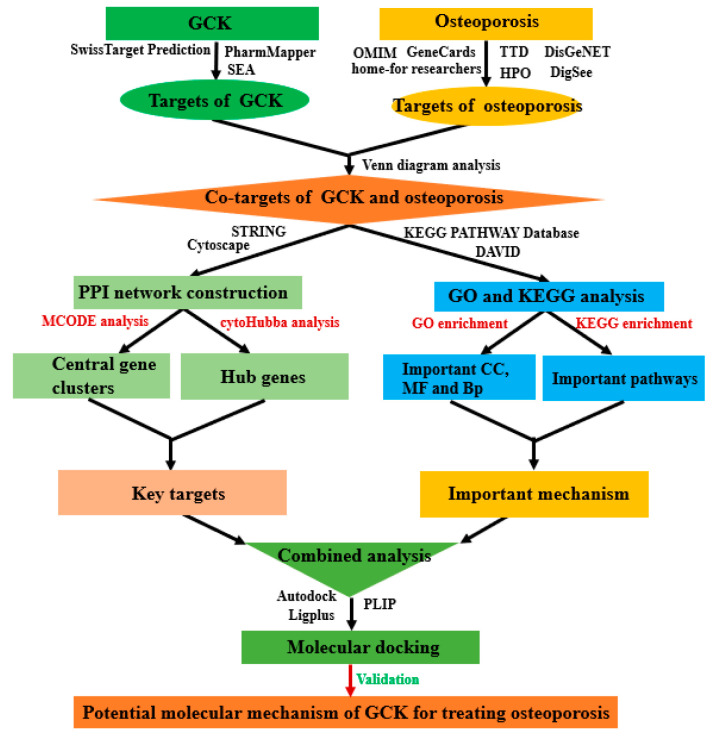
Workflow diagram of network pharmacology analysis of targets and molecular mechanism of GCK for treating osteoporosis.

Network pharmacology (NP) has recently been proposed as a promising approach to integrating database mining, bioinformatics analysis, topological analysis and molecular simulation, and is widely applied to discover potential medicinal ingredients from herbal medicines and to predict their possible pharmacology mechanism at the molecular level [17]. Given the above considerations, we have investigated the role of GCK in treating osteoporosis based on NP and have further explored the possible molecular mechanism in this study (a flowchart of this study is shown in Figure 1).

## 2. Results

### 2.1. Molecular Targets of GCK and Osteoporosis

**Figure 2:**
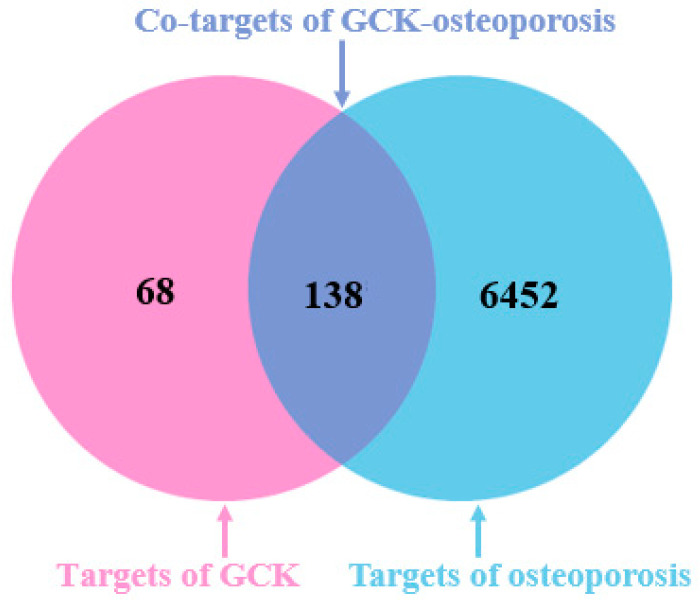
Co-targets of GCK–osteoporosis based on Venn diagram analysis. The red circle represents the targets of GCK, the blue circle represents the targets of osteoporosis, and the purple overlap represents the co-targets of GCK and osteoporosis.

A total of 206 and 6590 molecular targets were obtained for GCK and osteoporosis in this study, respectively. The overlapping targets of GCK and osteoporosis were considered as the potential targets of GCK-treated osteoporosis. Based on the intersection analysis, a total of 138 molecular targets were identified as co-targets of GCK and osteoporosis, and are shown in a Venn diagram (Figure 2).

### 2.2. PPI Network Analysis of Co-Targets of GCK and Osteoporosis

**Figure 3:**
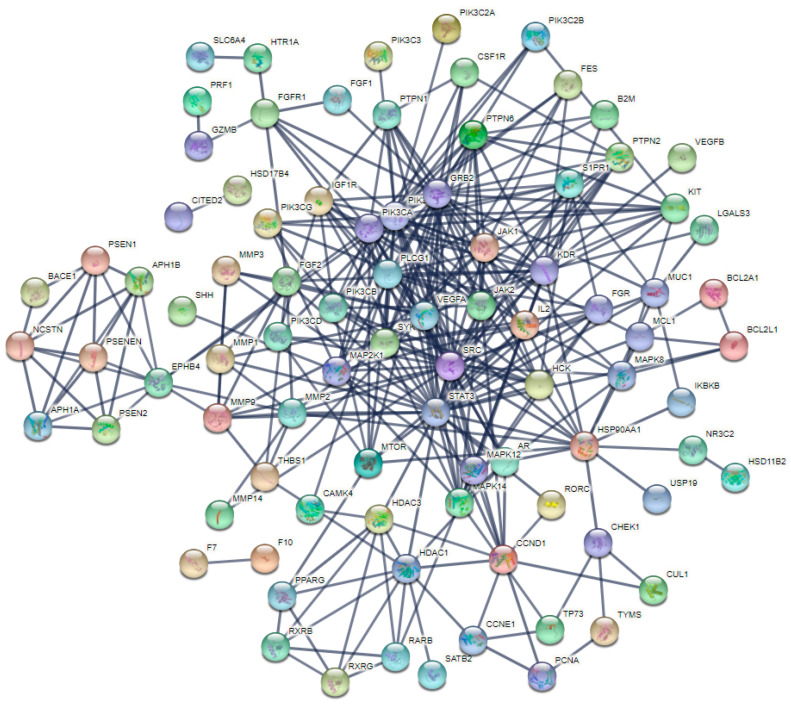
PPI network of the co-targets of GCK–osteoporosis constructed by STRING with the highest confidence score (0.9). Gray lines represent the interactions between the co-target proteins of GCK–osteoporosis, and the colored balls represent the different co-target proteins of GCK–osteoporosis.

**Figure 4:**
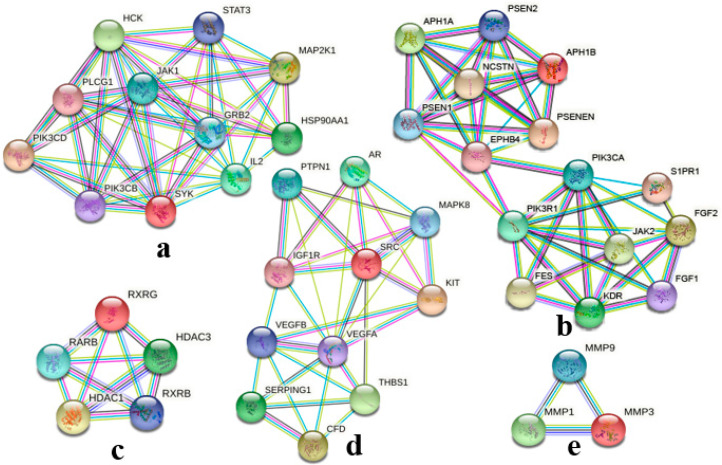
Central gene clusters identified in the GCK–osteoporosis PPI network based on MCODE analysis ((**a**), (**b**), (**c**), (**d**), (**e**) was cluster 1, 2, 3, 4 and 5, respectively.). Colored lines represent the evidence which supports the interaction between different target proteins and the colored balls represent the different target proteins.

**Figure 5:**
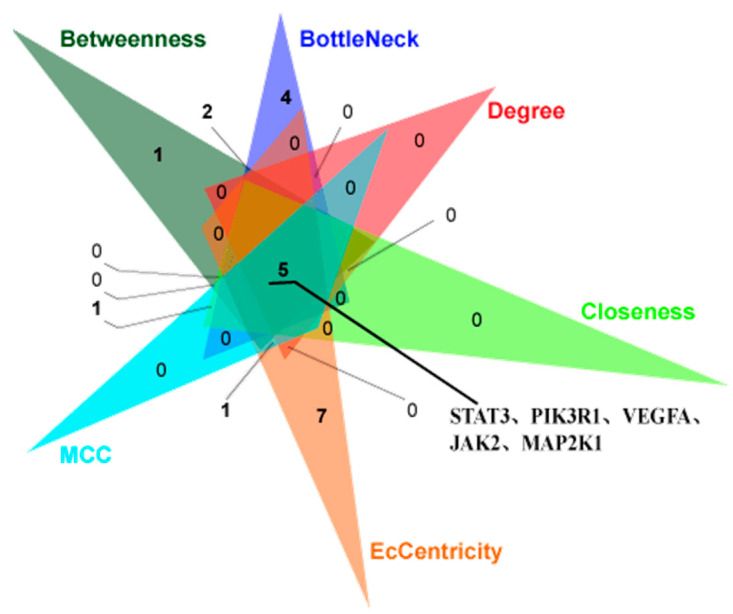
Key genes identified in GCK–osteoporosis PPI network based on Betweenness, BottleNeck, Degree, Closeness, MCC and EcCentricity method through the cytoHubba plugin of Cytoscape software 3.8.0. Triangles with different colors represent the top 20 target proteins screened in GCK–osteoporosis PPI network through the different topological methods. In this figure, “5” represents the overlap of proteins which were identified by all methods.

For investigating the internal connections and important targets of the co-targets of GCK and osteoporosis, the PPI network analysis was applied further here. After importing GCK–osteoporosis co-targets into STRING, we obtained the PPI network of the co-targets with the highest confidence (*p* ≥ 0.900), which contained 95 nodes and 365 edges (Figure 3). In this PPI network, the degrees of nodes PIK3R1, PIK3CA, STAT3, SRC, GRB2, PLCG1 and VEGFA were greater than or equal to 20, and were 35, 34, 30, 23, 22, 21 and 20, respectively. The target information of the PPI network queried from the STRING database was analyzed further by MCODE and the cytoHubba tool of Cytoscape software was used for identifying the key target proteins. MCODE analysis showed five central gene clusters (Figure 4 and Table 1). In these clusters, only the top one module returned a score > 6 in this PPI network (Figure 4a and Table 1), which contained SYK, STAT3, PLCG1, PIK3CD, PIK3CB, MAP2K1, JAK1, IL2, HSP90AA1, HCK and GRB2. Additionally, STAT3, PIK3R1, VEGFA, JAK2 and MAP2K1 were identified as key genes in the PPI network with the cytoHubba tool based on six forms of topological properties (Figure 5 and Table 2), which resulted in the three of the five central gene clusters identified by MCODE analysis (Figure 4a–c). This suggested that the related target molecules had an important role in GCK-treated osteoporosis, especially for the targets STAT3, PIK3R1, VEGFA, JAK2 and MAP2K1.

### 2.3. GO and KEGG Analysis of Co-Targets of GCK and Osteoporosis

**Figure 6:**
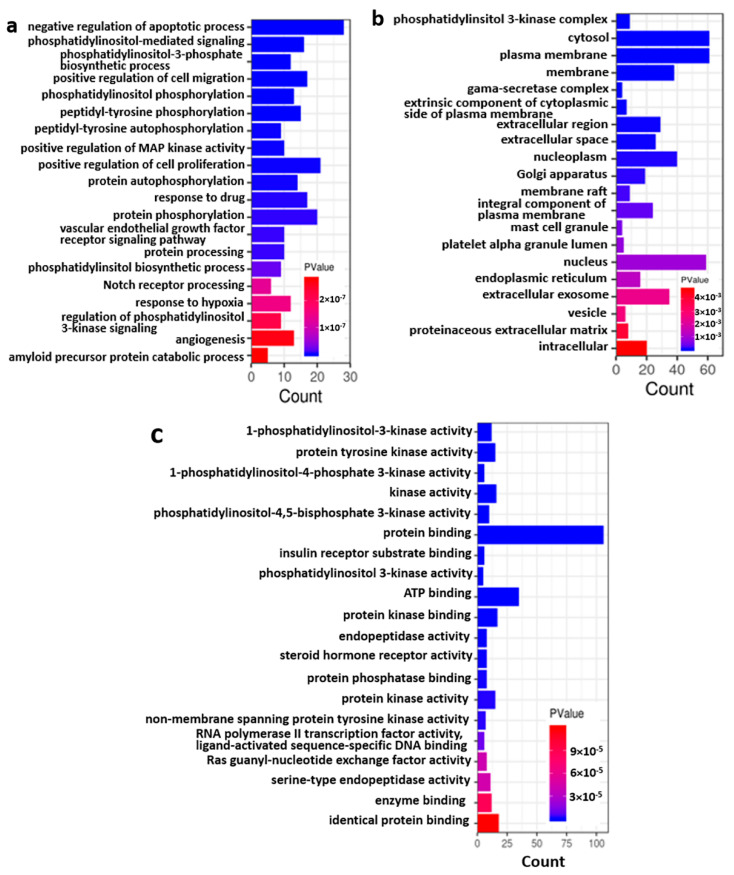
GO term enrichment for co-targets of GCK–osteoporosis ((**a**): enrichment of biological process; (**b**): enrichment of the cellular component; (**c**): enrichment of the molecular function).

GO and KEGG analysis were further used to investigate the BP, CC, MF and signaling pathways of the co-targets of GCK and osteoporosis. The results indicated that 210, 55 and 66 terms were enriched for BP, CC and MF, respectively. The top 20 BP, CC and MF terms (which were ranked based on *p* value) are shown in Figure 6a–c, respectively. The enriched BPs mainly included cell growth- and death-related processes (such as the negative regulation of apoptotic processes, the positive regulation of MAP kinase activity, the positive regulation of cell proliferation, the regulation of phosphatidylinositol 3-kinase signaling, and so on), protein synthetic and modification processes (such as phosphatidylinositol-3-phosphate biosynthetic process, phosphatidylinositol phosphorylation, peptidyl-tyrosine (auto)phosphorylation, protein (auto)phosphorylation, protein processing and so on ), and some stress response processes (such as drug, hypoxia, inflammatory and innate immune response). Based on the CC enrichment analysis, the co-targets were found to be mainly located in the plasma membrane, nuclear membrane, organelle, cytoplasm, extracellular matrix, extracellular secretion and exosomes, in which the number of target molecules from the membrane were more than the number of target molecules in the cytoplasm and extracellular components. In the MF enrichment analysis, the co-targets were mostly involved in kinase activity and protein/receptor/enzyme/small molecular-binding function. It is worth noting that the phosphatidylinositol-related biosynthetic process, protein modification, complex assembly and mediated signaling and kinase activities were among the top in the BP, CC and MF enrichment analyses. This suggests that the phosphatidylinositol-related bioprocess and signaling pathway might be one of the most important mechanisms for GCK-treated osteoporosis.

**Figure 7:**
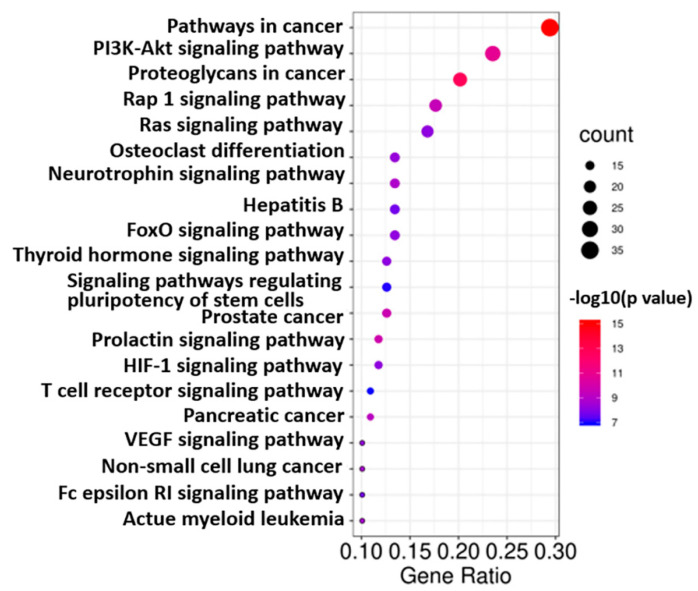
Top 20 pathways enriched through KEGG analysis for co-targets of GCK–osteoporosis.

**Figure 8:**
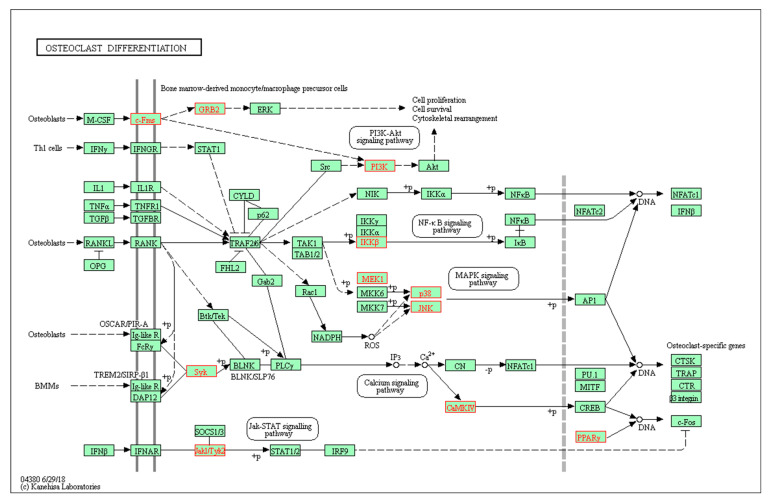
Osteoclast differentiation pathway obtained through KEGG enrichment analysis of co-targets of GCK–osteoporosis (all of the enriched targets are marked in red).

KEGG analysis showed that the co-targets of GCK and osteoporosis were mainly involved in 88 pathways (the top 20 pathways are shown in Figure 7), most of which were cancer-related pathways (such as pathways in cancer, proteoglycans in cancer, prostate cancer, pancreatic cancer, non-small cell lung cancer and so on). However, only the osteoclast differentiation pathway (shown in Figure 8) was significantly related to osteoporosis. In total, 16 genes (c-Fms, MAP2K1, SYK, PIK3CD, PIK3CB, PIK3R1, MAPK14, PIK3CG, MAPK12, IKBKB, MAPK8, PIK3CA, CAMK4, GRB2, PPARG and JAK1) were enriched in the osteoclast differentiation pathway with a *p* value of 4.81 × 10^−9^. In these 16 genes, just c-Fms was located in the cell membrane, and the others were located in the cytoplasm and nucleus. This indicated that c-Fms may play a more key role than other targets through regulating downstream signal transduction in the osteoclast differentiation pathway for GCK-treated osteoporosis.

### 2.4. Molecular Docking of GCK- c-Fms Interaction

**Figure 9:**
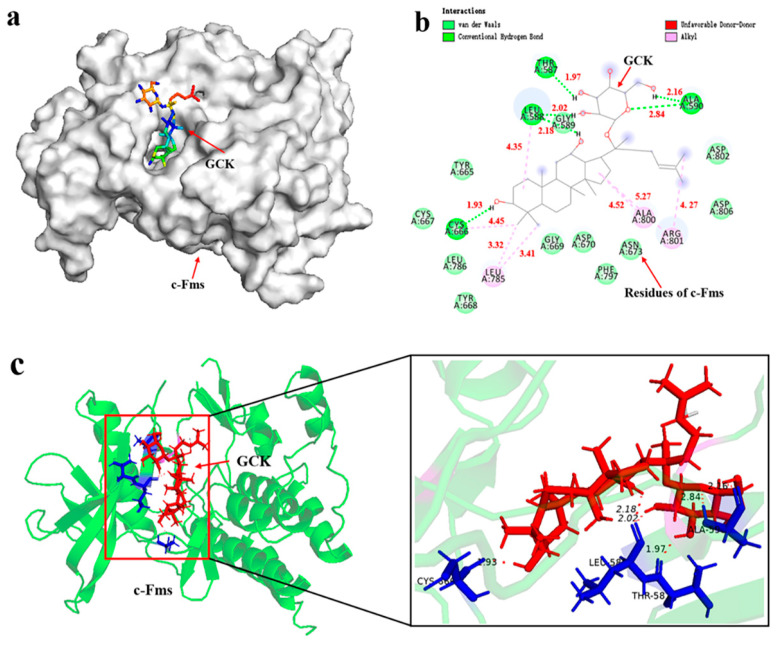
Molecular docking of c-Fms and GCK (**a**): diagram of molecular GCK located on the cavy of the c-Fms protein surface; (**b**): GCK-c-Fms interactions on a 2D diagram; (**c**): hydrogen bond interactions between GCK and amino acid residues of c-Fms on a 3D diagram.

Based on the results mentioned above, c-Fms-mediated signaling may be one of the most significant pathways, which could exert influence through interfering with osteoclast differentiation for GCK-treated osteoporosis. To validate the possible biological interactions between GCK and c-Fms, molecular docking was used here. The results showed that GCK could bind to the cavity on the surface of the c-Fms protein well with the lowest binding energy (−8.27 Kcal/mol) (Figure 9a). The complex of GCK-c-Fms was primarily stabilized by hydrogen bonds, van der Waals interactions and alkyl hydrophobic interactions (Figure 9b). Hydrogen bonds were formed between the GCK and c-Fms residues including Thr ^578^ (1.97 Å), Leu^588^ (2.02 Å, 2.18 Å), Ala^590^ (2.16 Å, 2.84 Å) and Cys ^666^ (1.93 Å) (Figure 9b,c), respectively. Alkyl hydrophobic interactions formed between GCK and the residues of Cys ^666^ (4.45 Å), Leu^785^ (3.32 Å, 3.41 Å), Ala^800^(4.52 Å) and Arg^801^(4.27 Å, 5.27 Å) of the c-Fms protein (Figure 9b). The van der Waals interactions were mediated by the surrounding hydrophobic pocket that formed Gly^589^, Tyr^665^, Cys^667^, Tyr^668^, Gly^669^, Asp^670^, Asn^673^, Leu^786^, Phe^797^, Asp^802^ and Asp^806^ (Figure 9b). Therefore, based on the results of molecular docking, GCK could interfere with osteoclast differentiation through interacting with the c-Fms protein, exerting its efficacy in the treatment of osteoporosis.

## 3. Discussion

Osteoporosis is a considerable clinical and public health burden because of its association with age-related fractures [2]. With the progressive aging of the global population, the incidence of osteoporosis will increase more than 5 times by 2050 around the world [18], which means that the population suffering from osteoporosis will sharply increase and exacerbate clinical and public health burden in the next 30 years. Therefore, it is vital to develop the drugs or functional food for the prevention and treatment of osteoporosis. Ginsenosides show promising potential for the prevention and treatment of age-related diseases including osteoporosis. Although the effects of ginsenosides on osteoporosis have been investigated for Rb1, Rb2, Rg1, Rg3 and Rh2, it is a pity that the efficacy of GCK on osteoporosis is unknown thus far, not to mention the molecular mechanism. In this study, we found that at least 138 molecular targets responded to osteoporosis and GCK simultaneously. Although these targets have not been obviously reported in other studies about ginsenoside-treated osteoporosis, some of them were verified indirectly based on the related-targets in the same pathways. For example, MAP2K1, MAPK12, MAPK14 and MAPK8 were related to MAPKs signaling which was trigged by Rb1 [19] and Rh2 [13], and mTOR was related to mTOR signaling which could be activated by Rg3 [12]. Consequently, this not only implies that GCK has the potential to prevent and treat osteoporosis, but also indicates that there is still a large unknown and a necessity to explore the molecular mechanism of ginsenoside-treated osteoporosis.

In this study, five osteoclast differentiation-related pathways (the PI3K-AKT signaling pathway, the NF-κB signaling pathway, the MAPK signaling pathway, the calcium signaling pathway and the Jak-STAT signaling pathway) were significantly enriched in KEGG analysis [13,19,20,21,22]. It was reported that ginsenosides had a poor permeability of cells with an apparent permeability coefficient of <1 × 10^−6^ cm/s [23,24]. Therefore, the membrane receptor-mediated signaling may be more efficient and important than the signaling proteins in the cytoplasm, which was also confirmed indirectly by the CC enrichment in the GO analysis. In this analysis of targets enriched in osteoclast differentiation-related pathways, c-Fms is the unique membrane receptor protein. Furthermore, we found that GCK could bind to the cavity on the surface of the c-Fms protein, which worked similarly to the small-molecule inhibitors of c-Fms [25,26]. The receptor-tyrosine kinase c-Fms (also known as CSF-1-R) is encoded by FMS or CSF-1-R proto-oncogene, which is the cell surface receptor for the (macrophage) colony-stimulating factor-1 (CSF-1 or M-CSF) [25]. c-Fms is expressed in macrophages, microglia, and osteoclasts is one type of receptor for M-CSF, and plays an important role in initiating inflammatory, cancer, and bone disorders when it binds with its ligand CSF [27]. Previous studies reported that the inhibition of c-Fms could prevent against osteoporosis by inhibiting osteoclast formation [27,28]. Additionally, the important structural motifs of c-Fms are the glycine-rich nucleotide-binding loop (residues 590–594), the activation loop (residues 796–825), the catalytic loop (residues 776–783), the native c-Fms kinase insert domain (residues 680–751) and the juxta-membrane domain (residues 538–581) [28]. In this study, we found GCK could bind to different residues of c-Fms through hydrogen bonds, van der Waals interactions and alkyl hydrophobic interactions. Especially, hydrogen bonds in residues Thr ^578^ (1.97 Å) and Ala^590^ (2.16 Å and 2.84 Å) were located in the juxta-membrane domain (which functions as an autoinhibitory region) and nucleotide-binding loop, respectively. Additionally, this was similar to quinolone (a small-molecule inhibitor of c-Fms was reported by Carsten [28]) for the interaction mode of GCK binding to c-Fms. Therefore, this suggests that GCK could inhibit the activity of c-Fms and c-Fms-related pathways through interactions with these residues, and finally inhibit osteoclast differentiation and formation.

Of the osteoclast differentiation-related pathways, the PI3K-AKT and MAPK signaling pathways were just to the downstream of c-Fms signaling. In addition, we found that phosphatidylinositol-related bioprocesses, proteins and signaling played an important role for GCK in the treatment of osteoporosis based on GO and KEGG enrichment analysis. This was especially true for PI3K-related signaling because subunit proteins of PI3K were included in the topped cluster of the PPI network and key genes which were verified with MCODE analysis and key gene analysis. Although it has not been reported for PI3K-related signaling in other studies about ginsenoside-treated osteoporosis, the PI3K/AKT pathway was considered to participate in anti-osteoporosis by promoting the proliferation of osteoblast precursors and the osteoblastic differentiation of BMSCs (bone marrow stromal cells), as well as autophagy and the differentiation of osteoclasts [20,29,30,31]. Meanwhile, in KEGG analysis, the PI3K/AKT pathway was enriched in the downstream of c-Fms-mediated osteoclast differentiation pathways. Therefore, GCK may inhibit osteoporosis through the inhibition of PI3K-mediated osteoclast differentiation. Although this is first reported for this molecular mechanism, regardless of research based on in vitro tests, in vivo tests and computing and simulation with NP approaches, this still needs to be validated further with more scientific evidence. We also note that cancer-related pathways were enriched at the top of the list in KEGG analysis for co-targets of GCK and osteoporosis. In previous studies, it has been verified that the PI3K pathway plays an important role in the bone metastasis of lung cancer and bladder cancer [32,33]. Therefore, it is suggested that GCK may also be involved in bone metastasis-induced osteoporosis through PI3K-related signaling in cancers.

In addition to phosphatidylinositol-related bioprocesses, proteins and signaling, GRB2-ERK was the other signaling pathway of c-Fms-mediated osteoclast differentiation. ERK is a member of the MAPK family which transduces extracellular stimuli to alter gene expression and has been shown to play a role in diverse cellular events ranging from proliferation and differentiation to apoptosis [34]. It was reported that M-CSF could activate ERK via the phosphorylation of c-Fms, which then recruits GRB2/SOS and stimulates the Ras/Raf/MEK(MAPK/ERK kinase)/ERK pathway [35]. Additionally, insulin may exert its anabolic effects on osteoblast through the IR-GRB2-ERK-mediated pathway [36]. Therefore, GCK may exert a similar or opposite effect on osteoclast differentiation through the regulation of the c-Fms-GRB2-ERK signaling axis. In addition to ERK, JNK and p38 of the MAPK family were also enriched downstream of the osteoclast differentiation pathway, and the MAPK-related protein MAP2K was verified to play a major role in GCK-treated osteoporosis in MCODE analysis and key gene analysis. These results suggest that GCK could activate the MAPK pathways involved in c-Fms-mediated downstream signaling in osteoclast differentiation. Although it has been reported that ginsenosides Rb1 and Rg3 could inhibit osteoclastogenesis and osteoclast differentiation by modulating the MAPK pathways [19,37], it was rarely reported that GCK could inhibit osteoclastogenesis and osteoclast differentiation by modulating Fms-mediated MAPK pathways. Therefore, it is necessary to validate these findings further with more evidence to identify whether GCK can inhibit osteoclast differentiation through the c-Fms-MAPK signaling axis.

**Figure 10:**
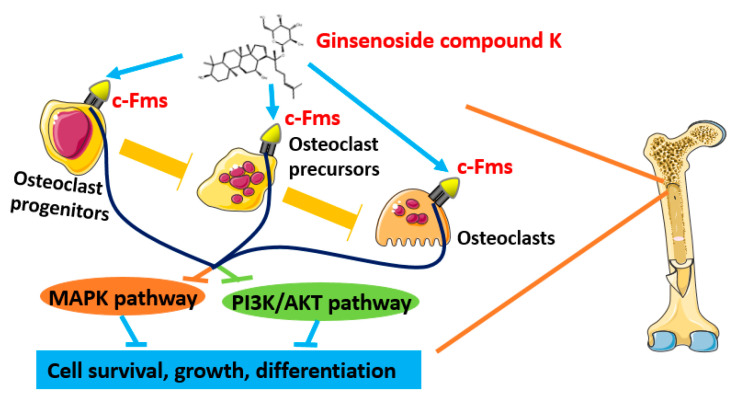
The putative schematic representation of the molecular mechanism of GCK in treating osteoporosis based on network pharmacology and in silico molecular docking.

Summarily, GCK has been verified to be potential beneficial in the treatment of osteoporosis based on NP in this study. Moreover, GCK could influence the occurrence and progress of osteoporosis through interacting with 138 potential target proteins at least, of which 16 targets were enriched in the osteoclast differentiation pathway based on KEGG analysis, and c-Fms-mediated osteoclast differentiation signaling may be one of the most important mechanisms for GCK in treating osteoporosis. Meanwhile, it was proven that GCK could bind to the cavity on the surface of c-Fms proteins with the lowest binding energy (−8.27 Kcal/moL) based on molecular docking. Additionally, PI3K and MAPK-related proteins were not only identified as important targets, but were also enriched in the downstream pathways of c-Fms-mediated osteoclast differentiation signaling. Therefore, we proposed that the c-Fms-mediated MAPK and PIK3 signaling axis may be the potential mechanism for GCK in the treatment of osteoporosis by interfering with osteoclast differentiation (an overview of the possible molecular mechanism is shown in Figure 10). These results are from rational and systematic computing and simulation on a chip based on the NP approach, but they still need to be deeply explored and validated with more in vitro and in vivo experiments, which will be focused on in the next work in our lab. 

## 4. Materials and Methods

### 4.1. Targets of GCK

The targets of GCK were predicted and obtained from PharmMapper (http://www.lilab-ecust.cn/pharmmapper/, accessed on 23 November 2020), Similarity Ensemble aApproach (SEA) (http://sea.bkslab.org/, accessed on 19 November 2020) and SwissTarget Prediction (http://www.swisstargetprediction.ch/, accessed on 20 November 2020) databases under the condition of Homo sapiens. Gene names and organisms were standardized through manual retrieval based on the Uniprot database (https://www.uniprot.org/, accessed on 25 November 2020) to avoid the over-annotation of similar proteins such as paralogs and putative products of pseudogenes.

### 4.2. Targets of Osteoporosis

The osteoporosis-related target proteins were obtained through online searches for “osteoporosis”, “fragile bones” and “bone fragility” under the condition of Homo sapiens in the following databases: GeneCards (https://www.genecards.org/, accessed on 18 November 2020), Online Mendelian Inheritance in Man (OMIM) (http://www.omim.org/, accessed on 18 November 2020), Therapeutic Target Database (TTD) (http://db.idrblab.net/ttd/, accessed on 18 November 2020), the Human Phenotype Ontology (HPO) (http://www.human-phenotype-ontology.org/, accessed on 18 November 2020), DisGeNET (http://www.disgenet.org/, accessed on 18 November 2020), DigSee (http://210.107.182.61/geneSearch/, accessed on 18 November 2020) and home-for-researchers (https://www.home-for-researchers.com/static/index.html#/project_assistant, accessed on 18 November 2020). The duplicate and redundant proteins or genes were deleted.

### 4.3. Putative Targets of GCK-Treated Osteoporosis

The overlapping targets of both GCK and osteoporosis were considered potential targets of GCK-treated osteoporosis, and were obtained through taking the intersection and Venn diagram analysis.

### 4.4. Protein–Protein Interactions (PPIs), Network Construction and Analysis

The intrinsic relationships between these putative targets of GCK-treated osteoporosis were analyzed further based on the STRING database (http://www.string-db.org/, accessed on 29 March 2021). The conditions of the PPI network construction were limited to “Homo sapiens” with the highest confidence score > 0.9. Furthermore, six kinds of topological properties (Betweenness, BottleNeck, Degree, Closeness, maximal clique centrality (MCC) and EcCentricity) of the PPI network were calculated to screen the key genes based on the topological importance using Cytoscape software (ver. 3.8.0) and the Cytoscape plugin cytoHubba [38]. The key genes were identified and obtained based on a Venn diagram analysis of the top 20 genes screened with different topological properties. Additionally, the Molecular Complex Detection (MCODE) module (a plug-in of Cytoscape) was used to identify the significantly enriched network clusters between node genes. In the MCODE analysis, degree cutoff value, node score cutoff value and K-Core value were set to 2, 0.2 and 2, respectively, and network clusters with an MCODE score ≥ 3 were screened as significantly enriched clusters.

### 4.5. Enrichment Analysis

Gene Ontology (GO) function and Kyoto Encyclopedia of Genes and Genomes (KEGG) pathway enrichment analyses were performed to reveal the potential biological mechanism of GCK in the treatment of osteoporosis. GO and KEGG enrichment were conducted through the online analysis tool of The Database for Annotation, Visualization and Integrated Discovery (DAVID, ver. 6.8) (https://david.ncifcrf.gov/, accessed on 29 March 2021) with *p* < 0.05. GO terms included three categories: biological process (BP), molecular function (MF), and cellular component (CC).

### 4.6. Molecular Docking of Compound–Target Interaction

Molecular docking was used to validate the potential mechanism of key proteins in osteoporosis-related pathways using Auto Dock software (ver. 1.5.6) [39] in this study. The 3D structure of the key target protein c-Fms (PDB 2I0V; protein length, 335; Homo sapiens) was obtained from the RCSB (Research Collaboratory for Structural Bioinformatics) PDB (Protein Data Bank) database (http://www.rcsb.org/, accessed on 20 November 2020). The 2D structure of GCK was downloaded from the PubChem database (https://pubchem.ncbi.nlm.nih.gov/, accessed on 20 November 2020) and its conformation of minimum energy was obtained through ChemOffice software (ver. 10) [40]. The protein and ligand were prepared using the AutoDock Tools prior to performing the docking process. The crystal structure of the target protein was pretreated, including the removal of water molecules (organic and heteroatom) and adding hydrogenation (charge and atom type). Auto Dock was utilized to semi-flexibly couple the GCK to the target protein with a genetic algorithm (GA), and the number of GA runs and the maximum number of evaluations were set to 200 and 2,500,000, respectively. Default values of Auto Dock software were used for other parameters of molecular docking.

## Figures and Tables

**Table 1:** Five central gene clusters of the GCK–osteoporosis PPI network identified based on MCODE analysis.

Cluster	Nodes	Edges	Score	Genes
1	11	36	7.200	SYK; STAT3; PLCG1; PIK3CD; PIK3CB; MAP2K1; JAK1; IL2; HSP90AA1; HCK; GRB2
2	15	40	5.714	S1PR1; PSENEN; PSEN2; PSEN1; PIK3R1; PIK3CA; NCSTN; KDR; JAK2; FGF2; FGF1; FES; EPHB4; APH1B; APH1A;
3	5	10	5.000	RXRG; RXRB; RARB; HDAC3; HDAC1
4	11	20	4.000	VEGFB; VEGFA; THBS1; SRC; SERPING1; PTPN1; MAPK8; KIT; IGF1R; CFD; AR
5	3	3	3.000	MMP9; MMP3; MMP1

**Table 2:** Top 20 key genes of the GCK–osteoporosis PPI network identified through the Betweenness, BottleNeck, Degree, Closeness, MCC and EcCentricity method based on cytoHubba tool.

Methods	Betweenness		BottleNeck		Degree		Closeness		MCC		EcCentricity	
	Gene	Score	Gene	Score	Gene	Score	Gene	Score	Gene	Score	Gene	Score
1	STAT3	1858.83	PIK3R1	32.00	PIK3R1	35.00	PIK3R1	60.00	PIK3R1	33,711.00	PIK3CA	0.32
2	PIK3R1	1514.88	STAT3	17.00	PIK3CA	34.00	PIK3CA	59.33	PIK3CA	33,710.00	PIK3R1	0.32
3	PIK3CA	1166.88	MMP9	12.00	STAT3	30.00	STAT3	57.08	GRB2	23,136.00	MMP9	0.24
4	EPHB4	1159.36	GRB2	9.00	SRC	23.00	SRC	52.00	PIK3CB	22,560.00	HCK	0.24
5	VEGFA	568.08	EPHB4	8.00	GRB2	22.00	GRB2	51.75	PLCG1	21,770.00	SYK	0.24
6	MTOR	477.35	VEGFA	7.00	PLCG1	21.00	VEGFA	50.17	JAK1	20,934.00	PTPN6	0.24
7	PLCG1	449.22	MAPK14	6.00	VEGFA	20.00	JAK2	49.67	SYK	17,220.00	IGF1R	0.24
8	CCND1	400.60	MTOR	4.00	JAK2	19.00	PLCG1	49.08	HCK	16,812.00	PIK3C2B	0.24
9	MAPK14	384.62	KIT	4.00	JAK1	19.00	JAK1	49.08	SRC	11,096.00	MAP2K1	0.24
10	JAK2	377.93	S1PR1	4.00	IL2	17.00	IL2	48.42	JAK2	10,906.00	STAT3	0.24
11	MMP2	328.71	PTPN6	3.00	PIK3CB	17.00	HSP90AA1	46.42	IL2	8767.00	MTOR	0.24
12	KIT	317.35	MAP2K1	3.00	SYK	15.00	PIK3CB	46.00	STAT3	7819.00	JAK2	0.24
13	IL2	299.91	JAK2	3.00	KDR	15.00	KDR	46.00	PIK3CD	7440.00	MMP1	0.24
14	SRC	296.80	PPARG	3.00	PTPN6	14.00	PTPN6	45.42	MAP2K1	5929.00	PIK3CD	0.24
15	MMP9	262.75	RARB	3.00	MAPK14	14.00	HCK	45.33	KDR	2580.00	MMP3	0.24
16	HDAC3	260.66	CHEK1	3.00	HCK	13.00	KIT	45.00	VEGFA	2392.00	VEGFA	0.24
17	S1PR1	237.33	MMP2	3.00	HSP90AA1	13.00	SYK	44.92	S1PR1	1538.00	KIT	0.24
18	GRB2	225.02	F10	3.00	MAP2K1	12.00	MAPK14	44.87	FGF2	1344.00	PTPN1	0.24
19	PTPN6	212.20	KDR	3.00	S1PR1	12.00	MAP2K1	44.75	HSP90AA1	1130.00	ANXA1	0.24
20	MAP2K1	205.76	HDAC3	3.00	MMP9	11.00	FGF2	44.75	EPHB4	724.00	GNRHR	0.24

## Data Availability

Not applicable.
